# The COP9 signalosome subunit 6 (CSN6): a potential oncogene

**DOI:** 10.1186/1747-1028-8-14

**Published:** 2013-11-28

**Authors:** Shang-Nuan Zhang, Dong-Sheng Pei, Jun-Nian Zheng

**Affiliations:** 1 Jiangsu Key Laboratory of Biological Cancer Therapy, Xuzhou Medical College, 84 West Huai-hai Road, Xuzhou, Jiangsu, P.R. China; 2 Center of Clinical Oncology, Affiliated Hospital of Xuzhou Medical College, Xuzhou 221002, China

**Keywords:** CSN, CSN6, Cancer, Ubiquitination, Deneddylation

## Abstract

CSN6 is one subunit of the constitutive photomorphogenesis 9 (COP9) signalosome (CSN), which is an evolutionarily conserved multiprotein complex found in plants and animals and originally described as a repressor of light-dependent growth and transcription in Arabidopsis. CSN is homologous to the 19S lid subcomplex of the 26S proteasome, thus it has been postulated to be a regulator of the ubiquitin-proteasome pathway. In mammalian cells, it consists of eight subunits (CSN1-CSN8). Among the CSN subunits, CSN5 and CSN6 are the only two that each contains an MPN (Mpr1p and Pad1p N-terminal) domain. The deneddylating activity of an MPN domain toward cullin-RING ubiquitin ligases (CRL) may coordinate CRL-mediated ubiquitination activity. More and more studies about CSN6 are emerging, and its overexpression is found in many types of cancers. Evidence has shown that CSN6 is a molecule platform between protein degradation and signal transduction. Here, we provide a summary of human CSN6, especially its roles in cancer, hoping that it can lay the groundwork for cancer prevention or therapy.

## Introduction

The COP9 signalosome, generally named CSN, is an evolutionarily conserved multiprotein complex existing in all eukaryotes. It consists of eight subunits termed CSN1-CSN8 [1]. Among the CSN subunits, CSN6 and CSN5 are the only two subunits that each share an MPN (Mpr1p and Pad1p N-terminal) domain, while other subunits contain a PCI domain, which may serve as a structure scaffold in the assembly of the COP9 signalosome [2-4].

The complex was originally described as a repressor of light-dependent growth in Arabidopsis by Deng and his collaborators [5]. CSN has diverse functions in cellular and developmental processes, including cell cycle control, signal transduction, transcriptional activation [6,7] and tumorigenesis [8,9]. The most and best studied function is regulation of ubiquitin-mediated protein degradation [10-13]. The function is fulfilled by removal of Nedd8/Rub1 (an ubiquitin-like molecule) from the cullin subunit of cullin-containing E3 ligases. And the deneddylation activity toward cullins is necessary for maintaining the stability and the sustained activity of cullin-RING E3 ligases (CRLs) in vivo, allowing the ligases to polyubiquitinate a large number of substrates that are targeted by the ubiquitin-proteasome system [14-17]. Since many key oncogene and tumor suppressor products such as p27 [18], c-Jun [19], p53 [8,20,21], COP1 [22] and 14-3-3σ [22] are degraded via the ubiquitin-proteasome pathway, it is conceivable that COP9 plays a significant role in cancer.

As a subunit of COP9 signalosome complex, CSN6 is found to be overexpressed in many types of cancers [8,23], linking it to oncogenic activity. Therefore, in this review, we provide an overview of the role of CSN6 in cancer and summarize recent findings that highlight the novel roles of CSN6 in cellular and developmental processes, suggesting that CSN6 is a promising therapeutic target in combating human cancers.

### CSN6 plays an important role in structural integrity of the CSN complex

The CSN is ~450 kDa in mass and comprises eight core subunits called CSN1–CSN8, in order of descending subunit size [24]. Then, how is this important complex organized structurally?

It has been shown that the complex is composed of two symmetrical modules, CSN1/2/3/8 and CSN4/5/6/7, connected by interactions between CSN1 and CSN6 [25]. As mentioned above, CSN6 and CSN5 are the only two subunits that each share an MPN (Mpr1p and Pad1p N-terminal) domain. Although CSN6 and CSN5 both contain the MPN domain, evidence has revealed that there are two types of MPN domains: one, like CSN5 with JAMM (Jab1/MPN/Mov34) motif, has the metalloprotease activity while another, the same as CSN6 without JAMM motif, has no related isopeptidase activity, which is probably involved in protein–protein interaction and in protein stability [26]. In fact, CSN6 contains an N-terminal MPN domain and a newly identified S6CD domain in the carboxyl half of the protein [27]. The MPN domain is responsible for the interaction with CSN5, and the S6CD-containing C-terminal half of CSN6 is in association with CSN4 and CSN7, suggesting that CSN6 is a core protein in the CSN4/5/6/7 subcomplex [28]. Moreover, earlier genetic studies in Arabidopsis showed that complete depletion of CSN6 resulted in loss of the entire CSN complex [29]. Therefore, CSN6 is essential for CSN assembly.

### CSN6 is involved in proteasome-mediated protein degradation

As mentioned above, COP9 coordinates CRL-mediated ubiquitination activity through its associated deneddylation activity toward the cullin subunit of cullin-containing E3 ligases. The deneddylation activity localizes to the JAMM (JAB1/MPN/Mov34) motif of CSN5 [30]. However, CSN5 alone has no metalloprotease activity unless it is associated with other subunits. Thus the deneddylation activity also requires the integrity of the CSN complex, which needs CSN6. Moreover, it has been shown that CSN6 makes a contribution to the binding of CSN and E3 ligases [30,31]. Recent study further demonstrates that the C-terminal region of CSN6 is necessary and sufficient for CSN complex integrity and recruitment of cullins to the CSN complex [27]. Taken together, CSN6 plays a pivotal role in proteasome-mediated protein degradation via regulating E3 ligases such as MDM2 [8] and COP1 [22].

### Roles of CSN6 in tumor

#### CSN6 is overexpressed in cancer

The human cancer patient transcriptomic data sets from Oncomine and Gene Expression Omnibus analyzed using Oncomine analysis tools and Nexus expression 2.0 reveal that many types of cancer have CSN6 overexpression, such as glioblastoma, breast cancer, myeloma, leukemia [23]. Zhao et al [8]. used the System for Integrative Genomic Microarray Analysis (SIGMA [32]) to evaluate genetic loss or gain of CSN6 (located at 7q22.1) using data from the British Columbia Cancer Agency Research (BCCRC) SMRT arrays, and found that a substantial percentage of samples (breast cancer cell lines and other types of cancer cells) had amplification of the CSN6 genomic region. And then they used quantitative PCR to confirm the gene amplification of CSN6 in breast cancer samples experimentally. Amplification of CSN6 was detected in a high percentage of breast cancer samples, and there was a positive correlation between CSN6 gene copy number and tumor size. Also, by comparing malignant follicular thyroid carcinomas with benign thyroid lesions (follicular adenomas, adenomatous nodules, and multinodular goiters) and normal thyroid tissue, they found that the carcinomas expressed higher levels of CSN6 than did benign lesions and tissues. These results suggest that CSN6 is overexpressed in cancer and is not restricted to a few specific types or cases of cancer.

#### Physiological significance of CSN6 overexpression in cancer

CSN6 is overexpressed in many types of cancer [8,23], linking it to oncogenic activity. However, detailed mechanisms through which CSN6 contributes toward carcinogenesis/tumor development remain unclear. Previously, human CSN6 was identified to interact with the human immunodeficiency virus 1 accessory protein Vpr (also called hVIP for Vpr interactive protein) and found to be involved in the G2/M phase transition of the cell cycle and cell proliferation [33]. Recently, more and more studies about CSN6 focus on the signaling pathways in which it is involved during carcinogenesis/tumor progression.

#### CSN6-MDM2-p53 axis

As a RING domain-containing E3 ubiquitin ligase, MDM2 can ubiquitin tumor suppressor p53 at several lysine residues, which causes p53 degradation. It can also degrade itself by autoubiquitination [34,35]. Overexpression of MDM2 is found in a wide variety of human tumors [36]. Zhao et al [8]. studied the expression of MDM2 and CSN6 in matched normal and cancerous breast tissues, and found that CSN6 was concomitantly overexpressed with MDM2 in human breast cancer tissues. Mechanism studies indicated that CSN6 prevented MDM2 autoubiquitination at lysine 364, resulting in stabilization of MDM2 and degradation of p53. Moreover, CSN6 couldn’t induce p53 degradation in Mdm2-null mouse embryonic fibroblasts, suggesting that CSN6-mediated degradation of p53 is MDM2 dependent. Mice in which CSN6 was deleted died early in embryogenesis, which could be rescued by concomitant loss of p53. Mice heterozygous for CSN6 (CSN6+/−) exposed to high doses of γ-irradiation (IR) showed more apoptosis due to an increased p53 activity. And loss of expression of CSN6 could attenuate carcinogenesis/tumor progression in response to DNA damage, which is known to be impeded by p53. These results suggest that CSN6 is an oncogene with positive activity toward MDM2 and plays a significant role in DNA damage-associated apoptosis and tumorigenesis through MDM2-p53 signaling pathway.

#### CSN6-COP1 axis

As another E3 ubiquitin ligase for p53, COP1 targets p53 for degradation by ubiquitin-dependent proteasome system, independently of MDM2 or Pirh2, which are known to interact with and negatively regulate p53, thereby maintaining p53 at low levels in unstressed cells and inhibiting p53-dependent transcription and apoptosis [37]. 14-3-3σ is a gene upregulated by p53 and has a positive feedback effect on p53 in response to DNA damage. It has been found to be frequently lost or decreased in various human cancers and functions as a potential tumor suppressor [38-40]. Recently, CSN6 was found to interact with COP1 and be involved in 14-3-3σ ubiquitin-mediated degradation [22]. Co-IP shows that CSN6 associates with COP1 endogenously and in vivo binding assay confirms that CSN6 directly binds to COP1 [22]. CSN6 stabilizes COP1 through reducing COP1 self-ubiquitination and decelerating COP1’s turnover rate [22]. The CSN6-COP1 axis has following physiological significance possibly: (1) CSN6 stabilizing COP1 directly enhances COP1-mediated p53 ubiquitination and degradation. (2) CSN6-COP1 axis causes 14-3-3σ degradation, which on the one hand, can block 14-3-3σ’s positive effect on p53 stability, on the other hand, activates Akt and promotes Akt-mediated cell survival.

#### CSN6-p57 axis

Recently, it is shown that CDK inhibitor (CDI) p57^kip2^ is a new target of CSN6 [41]. The p57^Kip2^ protein (abbreviated as p57) is a member of p21^Cip1^/p27^Kip1^ CDI family, sharing similar sequence with p27^Kip1^, also known as CDKN1C. Its overexpression causes a complete cell cycle arrest in G1 phase [42]. Decreased expression of p57 has been found in many types of cancer, including bladder carcinoma, gastric cancer and pancreatic cancer [43]. Stabilization of p57 is essential for the maintenance of its tumor suppressor function. Therefore, deregulation of proteins that affect p57 protein stability is expected to have an impact on human tumorigenesis. It’s well known that degradation of p57 is dependent on ubiquitylation, which is mediated by Skp2, an important component of Skp1/Cul1/F-box (SCF)-type E3 ubiquitin ligase [44]. Chen et al [41]. found that CSN6 was involved in p57 downregulation and increased the ubiquitination level of p57 in a dose-dependent manner. Mechanism studies show that CSN6 interacts with p57 and Skp2 through its C-terminal domain, which, in turn, promotes Skp2-mediated protein ubiquitination of p57, thereby decreasing the steady-state expression of p57. Significantly, CSN6 overexpression antagonized p57-mediated cell proliferation inhibition, G1 arrest and cell transformation suppression. Moreover, high expression levels of CSN6 and low expression levels of p57 correlated with poor overall survival in human tumor samples. Thereby, the CSN6-p57 link will be an important molecular target for cancer therapy and intervention [41].

#### HER2-Akt-CSN6 axis

CSN6 plays an important role in protein degradation, however, the molecular signals in regulating CSN6 activity are largely unknown. It is well known that HER2-Akt is critical in regulating p53 activity through MDM2 [45]. Recent study has shown that CSN6 is pivotal in regulating MDM2 to destabilize p53 [8]. Based on these studies, Lee’s laboratory further found that HER2-Akt axis is linked to CSN6 regulation, and that Akt is a positive regulator of CSN6 [46]. Akt is able to associate with CSN6 and phosphorylate it at Ser60, which can reduce the ubiquitin-mediated protein degradation and turnover rate of CSN6, thereby increasing steady-state expression of CSN6. Furthermore, Akt’s positive impact on CSN6 overexpression results in p53 degradation, cell transformation and DNA damage [46].

### CSN6 cleavage during apoptosis can regulate CSN-mediated deneddylation

In addition, few studies point out that CSN6 may participate in apoptosis process. For example, CSN6 can interact with amino-terminal caspase recruitment domain (CARD) of Nod1, a cytoplasmic protein that belongs to the Nod/NLR/CATERPILLER protein family and whose activation is involved in apoptotic pathways. During Nod1-induced apoptosis process, CSN6 is cleaved by recombinant caspase 8, suggesting that CSN6 is a direct target of caspase 8 [47]. However, how CSN6 is cleaved and the significance of CSN6 cleavage is still unclear. Later, in vitro and in vivo experiments further indicate that CSN6 is cleaved during apoptosis by activated caspases, most effectively by active caspase 3. And CSN6 cleavage is followed by cleavage of Rbx1, a component of CRL, thereby resulting in activation of CSN-mediated deneddylation and inactivation of CRLs [48]. These data demonstrate that CSN-mediated deneddylation can be regulated by active caspases and that the CSN6 executes a specific function during the apoptotic process.

## Concluding remarks

In this review, we have discussed the contribution of CSN6 to COP9 signalosome structurally and functionally, particularly focusing on roles of CSN6 in carcinogenesis/tumor development. As a subunit of COP9 signalosome, the roles of CSN6 in cancer could be linked to its involvement in ubiquitin-mediated protein degradation. However, the precise mechanism underlying its roles in regulating Cullin-RING E3 ligases remain to be fully elucidated.

It has been confirmed that deneddylation activity localizes to the JAMM (JAB1/MPN/Mov34) motif of CSN5 [30]. Nonetheless, the MPN domain of CSN6 is responsible for protein-protein interaction, while the newly identified S6CD domain in the carboxyl half of CSN6 is necessary and sufficient for CSN complex integrity and recruitment of cullins to the CSN complex [27], which is needed for deneddylation activity. Therefore, it seems that CSN6 executing its function is based on its effects on the whole CSN complex.

Recent findings about CSN6 mainly focus on its involvement in signaling pathways during tumorigenesis. For example, CSN6 connects HER2-Akt pathway with MDM2-p53 pathway [49], thereby regulating the expression of p53, a crucial tumor suppressor well known to us. In addition, CSN6 can stabilize COP1 and target substrates of COP1, such as 14-3-3σ and p53. Recently, p57, a new target of CSN6, is found to be regulated by CSN6 through Skp2. However, little is known about its upstream regulators. So far, few upstream regulators of CSN6 have been mentioned. For instance, Akt can positively regulate CSN6 by phosphorylation. Caspases also cleave CSN6 during apoptosis. Thus it is necessary to discover new regulators and targets of CSN6. As for the phosphorylation event, Akt can phosphorylate CSN6 and MDM2. However, could it regulate other p53 ubiquitin ligases, such as COP1, MDMx [50,51], to participate in p53 degradation? Is it also regulated by DNA damage? Could the upregulation of p53 in response to DNA damage have effect on CSN6 in turn?

In conclusion, CSN6 takes an active part in carcinogenesis/tumor development, suggesting its oncogene activity. Further investigations to understand the functions of CSN6 and its roles in cancer should be performed, thus significantly accelerating the development of a novel therapeutic strategy for various types of cancer.

## Competing interests

The authors declare that they have no competing interests.

## Authors’ contributions

SNZ collected and reviewed the literature and wrote the manuscript. DSP and JNZ corrected and revised the manuscript. All authors read and approved the final manuscript.
